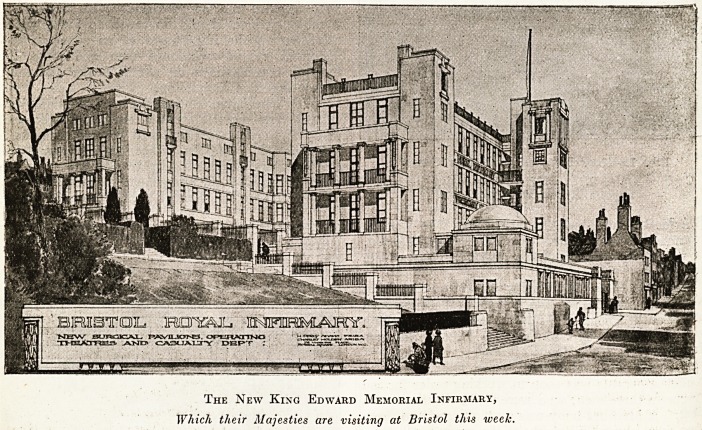# The King Edward Memorial Infirmary at Bristol

**Published:** 1912-06-29

**Authors:** 


### THE KING EDWARD MEMORIAL INFIRMARY AT BRISTOL.

### A Tour of the New Block with Mr. W. E. Budgett, Secretary and House Governor.

As soon as it was announced last May that the King, accompanied by the Queen, had consented to open the new Surgical Infirmary of 181 beds, which his Majesty had previously permitted to be called "The King Edward VII. Memorial Infirmary," our Commissioner visited Bristol, and through the courtesy of Mr. W. E. Budgett, Secretary and House Governor of the Bristol Royal Infirmary, was enabled to visit every part of the new buildings, the details of which Mr. Budgett personally explained.

### The New Infirmary from the Gardens.

After crossing the road from the old infirmary, Mr. Budgett led the way across planks and between workmen up the steep inclination of the hillside on which the Infirmary block has been built from the plans of Mr. Percy Adams, F.R.I.B.A., and Mr. Charles Holden, A.R.I.B.A., to various points in the beautiful gardens surrounding the nurses' home, from which the best views could be obtained, pointing out as he did so the dignified and severe design of the buildings. The way in which what had been a most difficult site had in a few months been transformed into grassy lawns, plantations of bamboos, interspersed with a very fine collection of flowering shrubs and plants was very remarkable.

"It is my ambition," Mr. Budgett explained, "to make the garden a point of attraction to our fellow-citizens, and already contributions from some of the most noted gardens in the West of England have been received, while part of the garden is laid out as a small nursery for raising plants from cuttings and seeds." Turning to the new Infirmary, he said: "You will see that the buildings are E shaped, the axis of the building running about north and south, the long arms of the E stretching east and west and containing twenty-four bed wards and their adjuncts, while the middle line of the E contains eight bed wards. Let us now begin a tour of the buildings."

### The Main Entrance and Casualty Department.

"How is the central quadrangle between the arms to be filled?"

"We are making a formal garden here on two levels, the upper stretching over the subway, which is open to the south and forms a colonnade shelter for the patients, being also the approach to that part of the garden set apart for the use of patients, the nurses' approach to their home being on the upper level." By this time we had reached the lower street level, and, entering the main entrance court, Mr. Budgett said: "You will see on the left the casualty entrance, at which ambulances will draw up protected from the gaze of curious crowds in the street by the outer gates, while directly in front of us is the main entrance door leading to the simple hall, between which and the casualty department is the porter's lodge and telephone-room. The porter is thus in complete control of both entrances. In this hall is the entrance to the subway which connects the old and new buildings. The casualty block lies below the whole length of the south arm of the E, and I will take you through this first. Here is the entrance hall, in which the friends of patients may

The New King Edward Memorial Infirmary,

*Which their Majesties are visiting at Bristol this week.*

wait. On the left is the casualty officer's room, and passing along the casualty corridor we come first to the room reserved for men, well lighted by windows as well as sky-lights, and opening out of it a top-lighted examination-room. On the other side the door leads to the casualty sister's room, which we pass on the way to the women's casualty room, which also has its private examination-room. Going out into the corridor again you will find the two and one bed observation wards, with convenient sink-room and lavatory near. Going back to the central hall we pass the casualty $x$-ray room."

"How many casualties a day are you now treating?"

"Nearly two hundred casualties and emergencies a day are treated, and we intend to admit our in-patients through these rooms, hence the size of the department."

"We will now suppose you are the patient who has been admitted. You will see there is an easy stretcher-way from the casualty department to the foot of the staircase and two large lifts which will convey you to the wards. One of these lifts is designed to carry a dozen people or two beds with their attendant nurses."

### The Treatment of the Stairs.

On passing the stairs, which are of terrazzo, Mr. Budgett called attention to the pains which had been taken in the design by which all the corners between the treads and risers had been coved and terrazzo carried over the outside string of the stairs to avoid the unsightly stains often caused by the trickling of dirty water over the edge to the ceiling below in the process of scrubbing. The well-hole is brightly lighted by a roof-light and large windows.

Here you are on the mezzanine floor, which will be used for special departments, and contains one eight-bed and one nine-bed ward, one small ward of two beds, two sanitary adjuncts, and bathroom.

Passing the next floor, which on account of the slope of the hill does not stretch over the whole area of the E, we enter that arm of the E which lies nearest the old Infirmary on the next complete floor, and, noticing our look of surprise at the size and length of the bare ward, for the workmen had only just finished it, Mr. Budgett said:

### A Typical Ward.

"This ward—and there are four others exactly similar in proportion—will contain twenty-four beds. The reason that this number was chosen is two-fold: in the first place, by it the cost per bed worked out most cheaply; and secondly, this number enabled us to use all the site, a problem which the site itself has made of some difficulty. As to the administration of a twenty-four-bedded ward, the matron, Miss Baillie, saw no difficulty in nursing a ward of this size, and so every consideration has been satisfied; the site, the need for a low cost per bed, and efficient administration. Now note some of the details. We will go round this ward, and you may take it as a type. As to lighting, there is a bed between each pair of windows, which, by the way, can be cleaned from the inside. The heating is obtained from two central stoves, which comprise four fire-places, and all the flues can be cleaned from the inside of the building. There is no ventilation contrivance except the windows, and there is not a concealed pipe about the place.

"The ward floors and window sills are of teak laid in bitumen; there is a central electric lighting and a bed light for every other bed, and there will be something which will startle you when the furnishing is completed. Every bed will be curtained. Its advantages, under proper supervision, are obvious. The bed itself, by the way, is a special design of our own. At the external extremity of the arm is a balcony, but you will be struck by the absence of sanitary towers. In their stead is the nurses' private lavatory and auxiliary sink-room, an arrangement by which the labour of nursing is considerably lightened, as we have placed the main sanitary tower at the internal end of the ward, and you will see it consists of a spacious bathroom, two closets, and large sink-room, to which we have devoted much attention. Here also is the external specimen cupboard. This tower is entirely cut off from the ward by a ventilated corridor, which opens through a door with louvre glass panels on to a small balcony, which will contain receptacles for dressings and dirty linen."

"What are the dimensions of the ward?"

"The ward is 102 feet long, 27 feet wide, and 13 feet 6 inches high, and there is a space of 8 feet 6 inches from centre to centre of each bed. Just inside the ward door is the sister's side-room on the right, and the entrance to a one-bed ward on the left. Passing out of the ward door we come to a two-bed ward, which can be served from the main ward through the one-bed ward, and on the other side of the corridor a large ward kitchen, with table, dresser, and sink. The stove is fitted in a recess lined with green tiles, and is heated by steam and gas. The kitchen floor is of red Ruabon tiles, which are turned for some inches up the walls. Opening out of the kitchen is a larder fitted with marble shelves and refrigerator. At the end of the main corridor is the ward linen-room, heated. There are two other wards exactly like this in the southern wing and two in the north, while in the centre are three eight-bed wards, with circular ends and the necessary adjuncts, some of which will be used for children. Opposite these on the main corridor are the patients' clothes rooms and scrubbers' closet. Each ward unit is entirely cut off by a bridge surrounded by air and with louvre windows."

### The Roof Ward and Roof Garden.

"We now go by the main staircase to the top floor of all, which consists of a roof garden over the south wing, which, of course, is of the same area as the ward immediately below. This will be used for nursing suitable patients, and you will notice that on the roof itself we have placed sink-room and lavatories for the nursing of patients here. The fire-escape staircase connects all the wards in this pavilion, and the lifts also come up here."

Here Mr. Budgett pointed out the extensive views to be obtained over the city and neighbourhood, which will probably make this one of the most popular points of the whole Infirmary.

### The New Theatres.

"How about the theatres themselves?"

"The theatres are on the top floor of the north wing, on a level with the flat roof over the centre and south wings. There are three altogether, with their anæsthetising rooms and students' rooms; but it is the way in which these divisions have been grouped on one floor and in relation to each other which is the really interesting thing. The third theatre at the extremity of the pavilion is a self-contained unit, but passing, as we are doing, from the back we come first to the surgeons' consulting-room. Next is the students' room and the theatre sisters' room, and close by a small room for pathological examinations. Then comes an anæsthetising room, then a theatre; then a wash-up room, where six persons can be accommodated. But before passing on to the next you will note that all the theatres can be approached from the corridor, and are also in communication at their window ends by a narrow passage for students near the theatre windows. On the outside of these, by the way, a balcony runs along, so that all discharge pipes and the windows themselves can be examined and cleaned without risk or inconvenience. This was my idea, and we think it will prove a useful addition. Beyond the wash-up room, which divides two theatres, are a small sterilising-room and the main instrument cupboard. Contiguous to this is the second students' entrance. Notice, in passing, the theatre nurses' lavatory and the surgeons' cloakroom, lavatory, and bathroom."

"The surgical block is very large?"

"Yes; and here it may be well to explain why. First of all, it is true, of course, that practically all the West of England sends its serious cases to Bristol, and the Royal Infirmary, even in the old building, has stood for years second to none in prestige in the West Country for surgery. But that is not the sole explanation. Bristol is not only a port but a university city, and in connection with the university there is a large students' school, the medical members of which look to the Royal Infirmary for their training. One point as to construction should be noticed. Here, as elsewhere throughout the new block, tiles and marble have been banished, and the general scheme of terrazzo floors and enamel alone has been carried out through the theatres."

### The Accommodation and the Scheme.

"What is the total accommodation of the King Edward VII. Memorial Infirmary?"

"It provides 181 beds distributed between five wards of twenty-four beds, three of eight beds, one of ten beds, one of nine beds, five of two beds, and five of one bed; three complete theatre units, casualty department, and entrance hall; whilst detached from the buildings on the north side is a large boiler-house, engineers' rooms, coal stores, and ash-yard. The boilers here heat the whole of the buildings and nurses' homes, and through steam calorifiers provide hot water for the whole Infirmary, both old and new. The steam and water pipes are conducted in subways over the whole site, and all the pipes are accessible.

"The story of the whole scheme, however, in its general outlines, was told in The Hospital of May 11, 1911, two months after the foundation-stone laying by our President, Sir George White, Bart., when you published an article on him in your 'Eminent Chairmen Series.'"

"Is there room for further extension?"

"I am glad you have mentioned this. The present buildings will cost between £70,000 and £80,000, but we hope this is only the first instalment of a scheme designed for the removal of all the patients from our old buildings by the erection of new blocks on our extensive site, so that our old buildings may be used for the housing of our servants and for the out-patient departments."

### The Question of Cost.

"What is the financial aspect?"

"It has been estimated to cost £70,000, of which we were able in May to announce that about half had been raised. But it is most important that the cost of the memorial should not be isolated, but considered in relation to the whole financial policy which was initiated by Sir George White when he was elected to the presidency of the Royal Infirmary in 1894. Our aim has been to increase the number of annual subscriptions, and our success has been very marked. Seven years ago this source of income amounted to £2,700 a year; by last year it had risen to £6,500, and, judging from the first four months of 1912, it is still increasing. My own work has lain in the field of increasing our income from this source; that is the keystone of our financial policy. Its coping stone and details depend upon this, and were publicly described by Sir George White a few weeks ago. You had better have his own explanation.

### Sir George White's Policy.

"In answer to the notion that that moiety of £70,000 which had not been subscribed for the completion of the building was a debt to be paid off, Sir George White explained our policy as follows:

"'I should like the public to understand (he said) that there is no question of a debt at all. This is not the usual case of the money having to be raised for the building. The invested funds will, so far as they are not supplemented by special donations, be used to meet the outlay. We have increased our subscription income by a sum largely in excess of any money which may be lost in consequence of the reduced return from investments. We call it a profitable transaction.

### A New Light on Hospital Finance.

"'If,' Sir George White argued, 'we have drawn temporarily upon our investment fund, we have done so as a matter of high policy. Our argument is that the bold course of adding 180 beds has been the means of increasing our income by the large amount of upwards of £3,000 a year, and the number of individual subscribers from 1,272 to 3,426.'

"Then he clinched the argument as follows:

"'After allowing for the loss of interest, on the balance which we have found for the memorial there is still a net increase of about £3,000, as our reward for the policy we have pursued. As donations come in they will serve to replace the investment money; but, after all, it is income which counts.'

"This is a new light on hospital finance, is it not? It is a bold but non-speculative policy. Like the buildings which it finances, it is exceedingly simple and attractive. It is, like them, the combination of science and imagination."

"Is there any other point to be emphasised?"

"For the sake of completeness you may add this. The Royal Infirmary is the base hospital of the Second Southern Military District. On the outbreak of war we have undertaken to receive up to 500 patients, and to be ready for the Army's call on our beds within twenty-four hours."

### "A Leader to be Safely Followed."

"The completion of the new block has been an immense work?"

"Yes; it began in despair at the hopelessness of attempting to deal piecemeal with the old Infirmary, and it is no prophecy to say that it is ending in triumph. The Governors, when they secured Sir George White as President, knew they had a leader who could be safely followed: the rare combination of a man of ideas and a man of action. Designs were thrown open to competition, and, as soon as Mr. H. Percy Adams had carried the day, we consulted together, heard the proposals of the medical staff, whose wishes have been consulted, and began work. From the first I was attracted by the problems and difficulties of the scheme, and when I, who had been a member of the committee for ten years, was asked if I would become Secretary and House Governor, I accepted, determined at all events to see the scheme through."

## Figures and Tables

**Figure f1:**